# Environmental and biological cues for spawning in the crown-of-thorns starfish

**DOI:** 10.1371/journal.pone.0173964

**Published:** 2017-03-29

**Authors:** Ciemon Frank Caballes, Morgan S. Pratchett

**Affiliations:** ARC Centre of Excellence for Coral Reef Studies, James Cook University, Townsville, Queensland, Australia; Gettysburg College, UNITED STATES

## Abstract

Sporadic outbreaks of the coral-eating crown-of-thorns starfish are likely to be due, at least in part, to spatial and temporal variation in reproductive and settlement success. For gonochoric and broadcast spawning species such as crown-of-thorns starfish, spawning synchrony is fundamental for achieving high rates of fertilization. Highly synchronized gamete release within and among distinct populations is typically the result of the entrainment of neurohormonal endogenous rhythms by cues from the environment. In this study, we conducted multiple spawning assays to test the effects of temperature change, reduced salinity and nutrient enrichment of seawater, phytoplankton, gametes (sperm and eggs), and the combined effect of sperm and phytoplankton on the likelihood of spawning in male and female crown-of-thorns starfish. We also investigated sex-specific responses to each of these potential spawning cues. We found that (1) abrupt temperature change (an increase of 4°C) induced spawning in males, but less so in females; (2) males often spawned in response to the presence of phytoplankton, but none of the females spawned in response to these cues; (3) the presence of sperm in the water column induced males and females to spawn, although additive and synergistic effects of sperm and phytoplankton were not significant; and (4) males are more sensitive to the spawning cues tested and most likely spawn prior to females. We propose that environmental cues act as spawning ‘inducers’ by causing the release of hormones (gonad stimulating substance) in sensitive males, while biological cues (pheromones) from released sperm, in turn, act as spawning ‘synchronizers’ by triggering a hormonal cascade resulting in gamete shedding by conspecifics. Given the immediate temporal linkage between the timing of spawning and fertilization events, variability in the extent and synchronicity of gamete release will significantly influence reproductive success and may account for fluctuations in the abundance of crown-of-thorns starfish.

## Introduction

Population outbreaks of the coral-eating crown-of-thorns starfish often result in extensive coral mortality [1] with highly extended recovery times [2], thereby contributing significantly to sustained and ongoing declines in coral cover across the Indo-Pacific. Given that crown-of-thorns starfish mature quickly (within two years [3]) and can have very high fecundity (>100 million oocytes per season for a single female starfish [4]) they are capable of very rapid increases in population size. However, densities of crown-of-thorns starfish vary enormously in space and time [5], pointing to major fluctuations in reproductive success. Despite being one of the most studied species in coral reef environments, rates of reproductive success (and variation therein) for crown-of-thorns starfish are virtually unknown. Previous studies have shown that variation in the number and arrangement of spawning individuals, as well as the prevailing flow conditions, dictate the local concentration of gametes [6–8]. However, the extent to which spawning is synchronized (within and among populations) is the most fundamental constraint on the fertilization success of broadcast spawning, gonochoric species [9–11], such as crown-of-thorns starfish [12,13].

Gametogenesis and spawning in asteroids is, in part, regulated by endogenous neurohormonal mechanisms [14]. Relaxin-like gonad-stimulating peptides [15] produced by supporting cells beneath the outer layer of starfish radial nerves induce the production of a maturation-inducing hormone, 1-methyladenine [16]. Production of 1-methyladenine in ovarian follicle cells around oocytes [17] and interstitial cells in testes [18] begins immediately upon detection of gonad-stimulating peptides. This maturation-inducing substance induces the breakdown of the follicular envelope and germinal vesicle of the oocyte, thereby leading to oocyte maturation and spawning of gametes by contraction of the gonad wall [16]. The timing of gamete release is the result of the entrainment of these often tightly programmed endogenous rhythms by cues from the environment.

Environmental factors influencing the course of reproductive events in echinoderms are complex and spawning has been correlated with changes in temperature, photoperiod, lunar cycles, salinity, food abundance, and phytoplankton concentrations [14,19]. Exact triggers of synchronous spawning in marine invertebrates are not well known, partly because of the challenges involved in identifying spawning cues [19]. Spawning may be synchronous at the scale of meta-populations, where spawning is likely influenced by regional cues (e.g., lunar cycle, day length, temperature), or at scale of local populations (“epidemic” spawning), where gametogenic cycles are likely influenced by generic cues (e.g., water temperature), but actual spawning is largely determined by very localized phenomena [9,12,19]. For crown-of-thorns starfish, synchronous spawning has been observed among dense aggregations of adults, but the timing appears very unpredictable and it is unknown to what extent spawning is synchronized across discrete populations [1,3]. Notably, there have not been any specific studies that test for spawning synchrony at the scale of meta-populations of crown-of-thorns starfish, which would be possible based on intensive sampling of reproductive condition at multiple locations. Furthermore, there have been reports that spawning by crown-of-thorns starfish coincides with spawning by other sympatric asteroids [12,20,21], suggesting that there might be general heterospecific cues that initiate spawning.

On Australia’s Great Barrier Reef (GBR), the peak spawning period of crown-of-thorns starfish (between November and February) has been deduced from changes in gonad index, gonad condition or histology of ovaries and testes, and changes in oocyte size frequency distribution (see Table 2 in Pratchett et al. [1]). However, proximate cues that trigger gamete release are difficult to infer from periodic sampling (often done monthly) and analysis of gonads [19]. Systematic observations of spontaneous spawning in the field has also provided valuable information on the spawning behavior of crown-of-thorns starfish and levels of synchrony in relation to prevailing environmental conditions [13,22,23]. However, observations of spawning of crown-of-thorns starfish in the field are rare. Inferring from the few *in situ* observations of spontaneous spawning by crown-of-thorns starfish [1], synchronous spawning occurs most often during the falling tide, around late afternoon to evening.

Chemoreception is well documented among asteroids, and despite the absence of a central ganglion in the asteroid nervous system, its radial symmetry and disk-like body covered with receptor units provide an ideal mechanism for gross chemosensory perception and simultaneous monitoring of stimulus intensity at different positions on its surface [24]. Unspecialized epithelial cells, innervated by a plexus of the ectoneural system, have been proposed to be receptive to a wide range of stimuli [25]. Sloan and Campbell [24] also described chemically mediated responses in the terminal or sensory tube feet. Pearse et al. [26] also suggested that asteroid ocelli might be involved in the detection of spawning cues. Previous studies on the foraging behavior of crown-of-thorns starfish have documented its chemosensory ability [27,28], which may allow them to likewise perceive potential spawning cues such as changes in seawater temperature and quality, exudates from phytoplankton, and pheromones from conspecific gametes. Babcock and Mundy [13] noticed that starfish that ultimately spawned at Davies Reef in the GBR were unusually active for two hours prior to spawning, which might indicate the time period over which starfish respond to environmental spawning cues.

Effective cues for synchronized spawning within and among distinct populations must be distinguishable from background environmental variation and might also be expected to indicate periods that will maximize fertilization rates and/ or larval survival [22,29,30]. The summer spawning season of crown-of-thorns starfish in the GBR have coincided with peak seawater temperatures [1,13], increased diurnal temperature range [31–33], reduced salinity and high nutrient input from heavy freshwater runoff during flood events [34–36], and elevated densities and changes in community structure of phytoplankton [37,38]. Spawning events in multiple echinoderm species have been reported to follow abrupt changes in temperature [39,40]. Although temperature appears to influence local gametogenic cycles in crown-of-thorns starfish (reviewed in Pratchett et al. [1]), there is currently no evidence that temperature (either absolute temperatures or rapid changes in temperature) induce spawning. Mass spawning events in some temperate species of chiton, mussels, and sea urchins have also been linked to peaks in phytoplankton abundance [41–43]. Phytoplankton blooms associated with high flow events, usually following cyclones have been documented in the GBR [38]. In marine invertebrates with planktotrophic larvae, such as crown-of-thorns starfish, larval survival is often strongly influenced by food availability [44], thus one critical advantage of phytoplankton as a spawning cue is ensuring that gamete release is timed when environmental conditions are favorable for larval development and survival. Conversely, flood events associated with phytoplankton blooms are often coupled with significant reductions in salinity [34,35], which may have maladaptive consequences for fertilization success and early development [45]. The role of peak abundance of larval food supply (phytoplankton) on spawning induction in tropical asteroids remains poorly understood [46]. Inter-individual chemical communication through sex pheromones from conspecific gametes has also been proposed in several marine invertebrates [19]. Spawning by one individual in an aggregation of the sea urchin, *Sphaerechinus granularis*, induced other conspecifics to spawn [47]. The presence of sperm in the water column has been experimentally demonstrated to induce spawning in sea urchins [42,46] and starfish [48,49]. Further studies suggested a synergistic relationship between sperm and phytoplankton cues, where spawning response depends on whether sea urchins have been in contact with phytoplankton or phytoplankton extracts [50]. Conversely, Reuter and Levitan [46] found that phytoplankton alone did not induce spawning, but when a phytoplankton cue was followed by the addition of sperm, response time to sperm was significantly reduced.

The purpose of this study was to experimentally test potential spawning cues for crown-of-thorns starfish. To the best of our knowledge, explicit tests of spawning cues have never been undertaken for crown-of-thorns starfish, potentially due to logistic challenges associated with experimenting with crown-of-thorns starfish. Alternatively, previous such studies may simply have never been published due to null results or inconclusive findings. In this study, we tested the effects of temperature change, reduced salinity and nutrient enrichment of seawater, phytoplankton, addition of spawned gametes (sperm and eggs), and the combined effect of sperm and phytoplankton on the likelihood of spawning in males versus females. Apart from determining the proximate cues for spawning, these experiments were intended to better understand sexual dimorphism in response to cues and establish whether males or females spawn first. Despite its importance in understanding the mechanisms of synchronous spawning in marine invertebrates [51,52], few studies have examined sex-specific responses to spawning cues.

## Methods

### Collection and maintenance of specimens

This study was carried out in strict compliance with the guidelines set out by James Cook University and the Lizard Island Research Station. Collection of crown-of-thorns starfish was conducted under Great Barrier Reef Marine Park Authority (GBRMPA) Permit No.G13/36401.1. Adult specimens of the Pacific crown-of-thorns starfish (*Acanthaster* cf. *solaris*), ranging from 250 to 350 mm diameter, were collected in late November 2014 from Unnamed Reef 14–133 (14° 55.147’ S, 145° 30.492’ E) located 15 nautical miles (28 km) south of Lizard Island, in the northern Great Barrier Reef, Australia. Average seawater temperature at the collection site during the time of collection was 27.66°C. Starfish were promptly transported to the Lizard Island Research Station and placed in a 5000-l round fiberglass tank and maintained at ambient conditions (28.30 ± 0.67°C; 35.46 ± 0.07 psu; pH 8.17 ± 0.01) with continuous flow of fresh seawater. Individuals that were damaged due to handling and/or prematurely spawning due to stress were immediately separated and not used in any experiments. Sexes were also separated, whereby sex identification was done by making a small incision on the proximal region of the arms to collect and examine gonad contents [3]. Ovary and testes lobes were placed in 1-methyladenine to check if starfish were ready to spawn. Incisions were allowed to heal and close off for three days prior to undertaking spawning experiments [53].

### Bioassays for spawning induction

Experiments were conducted from late November to early December 2014, which is the likely period of peak spawning of crown-of-thorns starfish on the GBR [13]. Five sets of experiments were conducted to quantify the spawning response of crown-of-thorns starfish to (1) temperature, (2) seawater enrichment, (3) phytoplankton species, (4) addition of spawned gametes, and (5) synergistic effects of gametes and phytoplankton. Starfish were individually placed in plastic aquaria with 50-l seawater in a closed system and provided with constant aeration. Experiments were conducted in shaded wet benches so sunlight from 1500 to 1800 hours was able to penetrate and amount of light was evenly distributed among aquaria. Each bioassay ran for 12 h, from 1500 hours to 0300 hours to coincide with the times of day when spontaneous spawning was previously observed in the GBR [1]. Average photoperiod during the experiments was 13 h. A visual examination of released gametes was done every 15 min and when gametes were released from gonopores along most arms it was scored as “spawned” and the time of spawning was recorded. All replicates were completely independent and each individual sea star was only tested in a single treatment (i.e. one sea star per aquarium for a given treatment condition). Sea stars that have been exposed to a given treatment were not reused for other experiments.

#### Experiment 1

Spawning response to ambient northern GBR summer temperature (28°C), moderate temperature change (28°C to 30°C), and abrupt temperature change (26°C to 30°C) were assessed for this bioassay. Starfish in plastic aquaria with 0.45-μm filtered seawater were allowed to adjust to initial temperatures (28°C, 28°C, 26°C) for 3 h prior to changing to final temperature settings. Temperature treatments in the closed recirculating system were set using aquarium chillers (Hailea, Guangdong, China) or heaters (Eheim Jäger, Deizisau, Germany) attached to digital temperature controllers (Aqua Logic Inc., CA, USA). Five independent replicates of each sex were used per treatment (N = 30).

#### Experiment 2

This was conducted as procedural control experiments to evaluate spawning response to filtered seawater (control), low-salinity filtered seawater, and nutrient-enriched filtered seawater. Controls were prepared by filtering seawater through a 0.2-μm filter (FSW) to exclude microalgae. For the low-salinity treatment (LS-FSW), filtered freshwater was added until salinity was down to 25 psu. Nutrient-enriched seawater (NE-FSW) was prepared by adding 2 ml of AlgaBoost™ f/2 medium (AusAqua Pty., Ltd., Wallaroo, Australia) to 20 l of 0.2-μm filtered seawater, which was devoid of phytoplankton. Natural phytoplankton blooms are likely to be associated with reduced salinity and high nutrient inputs [38]. This experiment isolated the effects of salinity and nutrients from phytoplankton. Eight independent replicates of each sex were used per treatment (N = 48).

#### Experiment 3

This bioassay was used to test spawning response to monocultures of three species of common marine phytoplankton: the dinoflagellate *Dunaliella tertiolecta* (strain CS-175), and the diatoms *Skeletonema pseudocostatum* (strain CS-252) and *Chaetoceros muelleri* (CS-176). Axenic strains of microalgae were supplied by the Australian National Algae Culture Collection (CSIRO, Hobart, Tasmania). Monospecific cultures were maintained in exponential growth with the use of 0.2-μm filtered seawater enriched with AlgaBoost™ f/2 medium. The cultures were grown at 20°C under a 16-hour light: 8-hour dark cycle (daylight fluorescent lighting). Air filtered at 0.2-μm was continuously bubbled through the cultures. Sodium metasilicate pentahydrate (13 mg l^-1^) was added to seawater medium used to culture diatoms. Cell density was quantified daily using a haemocytometer. Concentrated cultures were placed in sealed glass bottles and allowed to sit in a water bath set at 28°C for 3 h before being added to each aquarium to reach a final concentration of 5 x 10^8^ cells l^-1^, based on previous spawning induction experiments on sea urchins [42,46]. Filtered seawater (FSW) was used for controls and eight independent replicates of each sex were used per treatment (N = 64).

#### Experiment 4

This bioassay was conducted to examine the spawning response of crown-of-thorns starfish to conspecific gametes. Eggs were collected from two female starfish induced to spawn by injecting 1 x 10^−4^ M 1-methyladenine on each arm junction 90 min before the experiment started. Eggs were transferred to clear containers with FSW and the number of eggs per mL was counted using a gridded slide under a dissecting microscope. Eggs were added to aquaria to achieve a final concentration of ~2 eggs ml^-1^. Sperm was collected from males 15 min before the experiment started using the same method employed for females above. Sperm concentration was quantified by haemocytometer counts and added to aquaria to achieve a concentration of 1 x 10^4^ sperm ml^-1^ [54]. Filtered seawater (FSW) used for controls was devoid of gametes and eight independent replicates of each sex were used per treatment (N = 48).

#### Experiment 5

This experiment was performed to determine whether sperm and high phytoplankton concentrations had a synergistic or additive effect on spawning response in crown-of-thorns starfish. It was not possible to test for synergies across all combinations of potential spawning cues (due to limitations in aquarium space and the number of starfish that could be housed), and this synergy was prioritized based on previous studies showing evidence of synergism between sperm and phytoplankton [42,46,50]; as well as limited evidence for threshold temperature and salinity [19]. Seawater (FSW) in control aquaria had no gametes or phytoplankton, while sperm treatments were the same as above. For sperm and phytoplankton (PP) treatments, a mixture of three species of phytoplankton (*D*. *tertiolecta*, *S*. *pseudocostatum*, *C*. *muelleri*), each at a concentration of 1.67 x 10^8^ cells ml^-1^, was added to the sperm suspension. Eight independent replicates of each sex were used per treatment (N = 48).

### Statistical analyses

The number of starfish that spawned in response to different treatments was arranged as a Model II contingency table, where marginal totals for each treatment (replicates) were fixed [55]. Contingency tables for each set of experiments were analyzed using log-linear models with log link and Poisson error terms [56] to examine the spawning response of crown-of-thorns starfish in relation to ‘Sex’ and ‘Treatment’. Spawning response was considered a response variable so all models included the interaction between ‘Sex’ and ‘Treatment’ [55]. Deviance statistics (G_2_) were used to compare models in R [57]. Odds ratio (OR) calculations for cells with zero observed counts were corrected by adding 0.5 to each cell [56]. Asymptotic standard errors were also obtained to calculate 95% confidence intervals for odds ratios. Pairwise comparisons were done using Fisher’s Exact Test implemented in R [57]. Distributions of spawning response time after exposure to independent treatments were compared using the Log-rank test, which is a widely used non-parametric test to compare time-to-event (time until spawning from initial treatment) distributions, while adjusting for right-censoring (termination of experiment after 12 h) [58]. This was followed by Holm-Šídák *post hoc* multiple comparisons (α = 0.05) implemented in Sigmaplot 12 (Systat Software, Inc., CA, USA).

## Results

### Effects of threshold temperature versus temperature change

**Fig 1:**
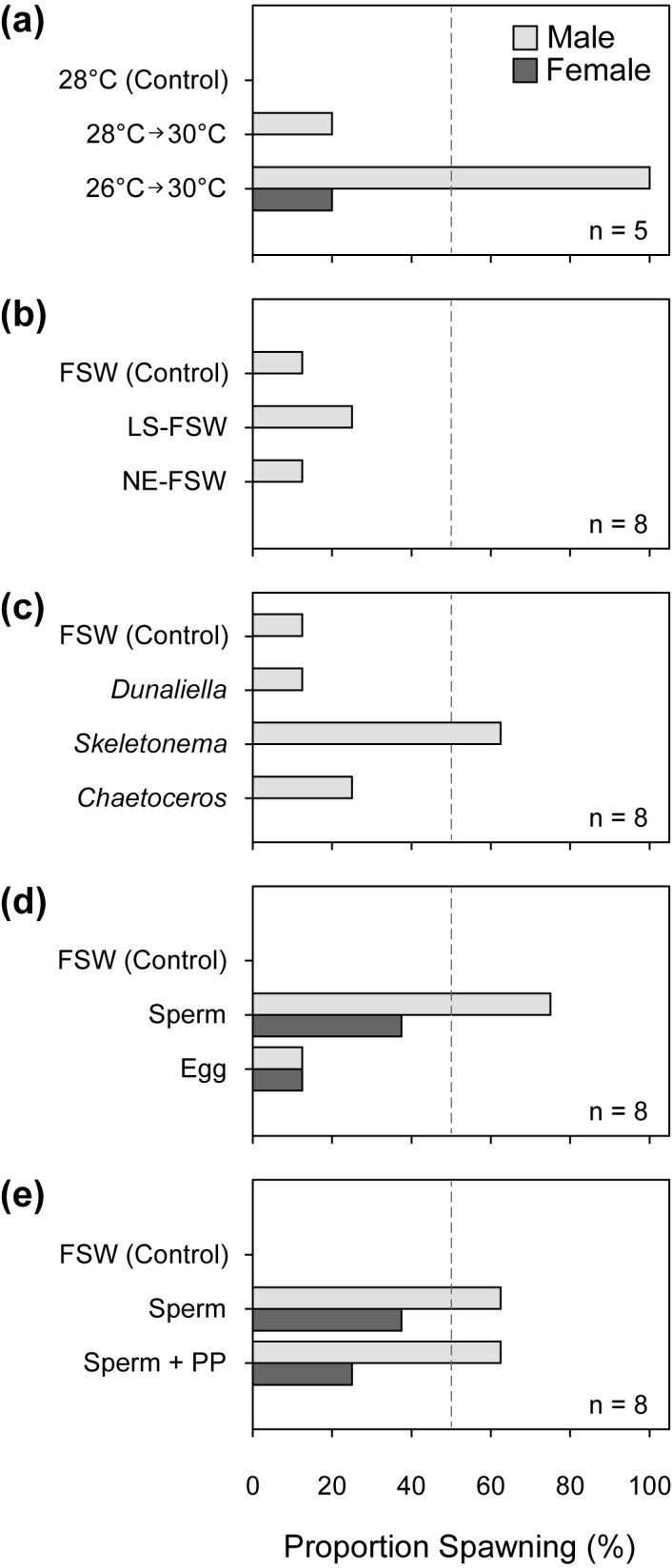
Proportion of starfish that spawned in response to cues. (**a**) seawater temperature, (**b**) water quality, (**c**) phytoplankton, (**d**) conspecific gametes, (**e**) sperm and phytoplankton. FSW = 0.2-μm filtered seawater; LS-FSW = low salinity filtered seawater; NE-FSW = nutrient-enriched filtered seawater; PP = combination of three phytoplankton species. Bars traversing the dashed lines represent spawning of more than 50% of individuals exposed to a given treatment.

**Fig 2:**
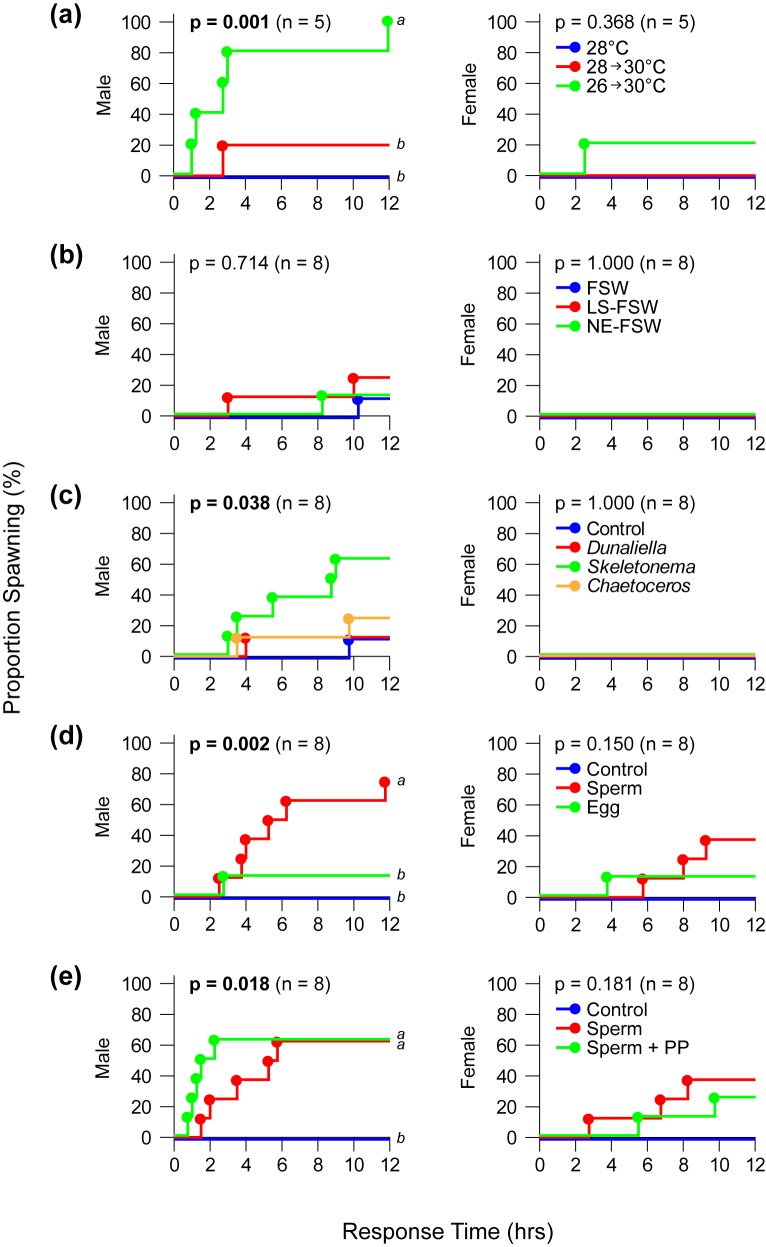
Response time and cumulative probability of spawning in male and female crown-of-thorns starfish after exposure to environmental and biological cues. (**a**) seawater temperature, (**b**) water quality, (**c**) phytoplankton, (**d**) conspecific gametes, (**e**) sperm and phytoplankton. Solid circles are individual spawning events and different letters indicate significant differences based on multiple comparisons (Holm-Šídák) after Log-rank analyses. FSW = 0.2-μm filtered seawater; LS-FSW = low salinity filtered seawater; NE-FSW = nutrient-enriched filtered seawater; PP = combination of three phytoplankton species.

Across all treatments, 40% of all males spawned compared to only 6.7% of female starfish (G_2_ = 9.954, df = 3, p = 0.019). Spawning response was found to be dependent on temperature change treatments (G_2_ = 17.530, df = 4, p = 0.002), where a +4°C temperature shock (26°C to 30°C) resulted in significantly higher spawning frequency in males (100%) compared to control (0%; OR = 121.000, 95% CI 2.017–7259.723) and +2°C temperature change treatment (20%; OR = 33.000, 95% CI 1.064–1023.620) treatments (**Fig 1A**). Females did not spawn under ‘no change’ and ‘moderate change’ treatments, and only spawned at a single instance when exposed to a +4°C temperature shock. Male spawning response time distribution (**Fig 2A**) was also significantly different among treatments (Log-rank χ^2^ = 8.623, df = 2, p = 0.013), but there was no significant treatment effect on time-to-spawning in female starfish (Log-rank χ^2^ = 2.000, df = 2, p = 0.368; **Fig 2A**). Male starfish spawned 240 min (±SE = 122 min) after exposure to temperature change from 26°C to 30°C. All log-linear model comparisons to test for complete dependence and conditional dependence for this experiment and subsequent experiments are summarized in **Table 1**. Odds ratios and 95% confidence intervals for pairwise comparisons of all treatments tested in each experiment are listed in **S1 Table**.

**Table 1:** Analysis of deviance table for hierarchical comparisons of log-linear models to test for patterns of complete dependence and conditional independence of variables inducing spawning response in crown-of-thorns starfish. ‘Spawning’ was considered to be a response variable, so all fitted models included the ‘Treatment’ by ‘Sex’ interaction term.

Source	G_2_	df	p-value
**(a)** *Temperature*			
Treatment	17.530	4	**0.002**
Sex	9.954	3	**0.019**
Treatment × Sex	3.400e-10	2	1.000
**(b)** *Water Quality*			
Treatment	0.573	4	0.966
Sex	5.965	3	0.113
Treatment × Sex	4.887e-10	2	1.000
**(c)** *Phytoplankton (PP)*			
Treatment	10.379	6	0.110
Sex	13.802	4	**0.008**
Treatment × Sex	3.307e-10	3	1.000
**(d)** *Gamete*			
Treatment	18.962	4	**0.001**
Sex	2.348	3	0.503
Treatment × Sex	0.736	2	0.692
**(e)** *Sperm + PP*			
Treatment	16.412	4	**0.003**
Sex	3.358	3	0.340
Treatment × Sex	0.153	2	0.926

### Effects of water quality properties

FSW control, low-salinity FSW, and nutrient-enriched FSW were ineffective in inducing high rates of spawning in crown-of-thorns starfish (**Table 1**). Spawning was not dependent on procedural treatments (G_2_ = 0.395, df = 4, p = 0.983), but an association with ‘Sex’ exists (G_2_ = 10.976, df = 3, p = 0.012), as 16.7% of males spawned and none of the female starfish spawned under all the treatments (**Fig 1B**). Only 12.5% of males spawned under FSW control and nutrient-enriched FSW treatments, and only 25% spawned under low-salinity FSW. For the males that spawned, response time distributions were also not significantly different from controls (Log-rank χ^2^ = 0.567, df = 2, p = 0.714; **Fig 2B**).

### Effects of phytoplankton monocultures

The incidence of spawning when crown-of-thorns starfish were exposed to monocultures of phytoplankton are significantly higher among males (37.5%; OR = 26.277, 95% CI 1.456–474.208) compared to females, where no spawning was observed across all treatments (G_2_ = 13.802, df = 4, p = 0.008). Among the phytoplankton species tested, exposure of males to *S*. *pseudocostatum* resulted in the highest frequency of spawning (62.5%), but was not significantly different from controls (12.5%) and other phytoplankton species (*Dunaliella*: 12.5%; *Chaetoceros* 25%) (G_2_ = 10.379, df = 6, p = 0.110; **Fig 1C**). Overall, different phytoplankton taxa had a significant effect on spawning response time distributions (Log-rank χ^2^ = 8.440, df = 3, p = 0.038), but none of the pairwise comparisons had enough power to meet the Holm-Šídák criterion (**Fig 2C**).

### Effects of conspecific gametes

Regardless of sex (G_2_ = 4.186, df = 3, p = 0.242), there was a significant increase in the incidence of spawning following addition of gametes (G_2_ = 17.008, df = 4, p = 0.002). The presence of sperm in the water column induced 75.0% of males and 37.5% of females to spawn, while only 12.5% of males and females spawned when exposed to eggs (**Fig 1D**). None of the starfish spawned under ‘Controls’. Among males, the incidence of spawning when exposed to sperm was 13 (95% CI 1.329–127.168) times higher than when exposed to eggs. Moreover, there was a significant difference in the cumulative probability of spawning and response times of males (Log-rank χ^2^ = 12.887, df = 2, p = 0.002); in particular, spawning rates (incidence and response time) were significantly higher in response to sperm compared to eggs and controls (**Fig 2D**). There was no significant difference in female spawning response time (Log-rank χ^2^ = 2.050, df = 2, p = 0.150) among gamete treatments (**Fig 2D**).

### Sperm and phytoplankton

Experiments to test the synergistic effects of phytoplankton and sperm showed that spawning response was dependent on ‘Treatment’ (G_2_ = 16.412, df = 4, p = 0.003) for both males and females. Sperm (50%) and phytoplankton-and-sperm (43.8%) treatments did not differ significantly, but spawning frequencies of males and females under both treatments were significantly higher than controls (**Fig 1E**). There was an overall difference in male spawning rates among treatments (Log-rank χ^2^ = 7.984, df = 2, p = 0.018), as response time under sperm and phytoplankton-and-sperm treatments was significantly faster compared to controls (**Fig 2E**). Although spawning response time between sperm and phytoplankton-and-sperm treatments did not differ significantly, starfish exposed to phytoplankton-and-sperm had shorter average response times (81 ± 15 mins) compared to sperm treatments (216 ± 51 mins). There was no difference in female response time among treatments (Log-rank χ^2^ = 4.277, df = 2, p = 0.181; **Fig 2E**).

## Discussion

While there have been no explicit tests of spawning cues for crown-of-thorns starfish, geographical differences in gametogenic cycles (reviewed by Pratchett et al. [1]) suggest that temperature is an important determinant of seasonal maturation, if not actual spawning. Temperature has been one of the most discussed potential spawning cues in the extensive literature available for marine invertebrates; despite this, very few studies have provided convincing evidence on the proximal role of temperature in gamete discharge [19]. In several echinoderm species, including crown-of-thorns starfish in the GBR, gametogenesis is clearly linked to local temperature regimes, but few studies have shown that specific changes in temperature or absolute temperatures stimulate gamete release (e.g. [59,60]). On the GBR, the long-term average sea surface temperature during the annual summer spawning season (mid November to mid January) is 28.00 ± 0.5°C. In our spawning experiments none of the gravid starfish spawned when maintained at 28°C, suggesting that threshold temperatures are not sufficient in their own right to induce spawning. However, gamete release in male crown-of-thorns starfish was triggered by an abrupt increase in seawater temperature, independent of any changes in nutrient concentrations, phytoplankton abundance, photoperiod, or conspecific interactions. Sea surface temperatures can vary > 4°C throughout the summer spawning season on the GBR, but within the course of a single day, temperatures usually vary within 1°C in the relatively deeper reef slope and within 1–2°C in the shallower reef flats [31]. Although rare, abrupt temperature changes have been reported in some parts of the GBR [32,33,61]. Temperature spikes from normal diurnal temperature variation have been associated with intense summer upwelling events in the GBR [61]. On a number of occasions, temperature change at a rate of 1°C per hour over a 6-hour period have been documented in inshore reefs around Magnetic Island. Diurnal temperature variation was usually more pronounced in reef flats, and varied on average by 4°C at more offshore reefs around Heron Island [33], but can vary by up to 5–7°C when tidal range is at its maximum [32]. Spawning observations of crown-of-thorns starfish *in vivo* have mostly been reported in shallow depths, where changes in seawater temperature are likely to be greatest. Babcock & Mundy [13] reported that all spawning starfish were found between 1 and 4 m deep during the spawning event of crown-of-thorns starfish observed at Davies Reef on the GBR. Although not very common in tropical reefs, these rapid increases in temperature may be important in triggering spawning.

Minchin [62] suggested that rapid increases in seawater temperature, caused by local moderate onshore winds on sunny days, induced spawning in the starfish *Marthasterias glacialis* in shallow waters (< 4-m depth). Himmelman et al. [40] reported that the mass spawning of several echinoderm species off the Mingan Islands in northern Gulf of St. Lawrence (eastern Canada) coincided with sharply increasing seawater temperatures brought by the incursion of warm surface waters. In natural settings, abrupt fluctuations in temperature may also result in alterations of seawater chemistry and may be associated with increased abundance of phytoplankton. Fine scale monitoring of concurrent environmental data during natural spawning events is needed to provide conclusive evidence for the role of temperature in gamete release.

Flood plumes in the GBR are characterized by medium to low salinity, high nutrient levels, increased chlorophyll-*a* concentration, and elevated phytoplankton abundance [38]. Reduced salinity and elevated nutrient levels did not induce gamete release in females and spawning frequency and response time in males was not significantly different from controls. Evidence for the role of salinity in spawning induction in echinoderms is scant and salinity fluctuations are typically minimal and short-lived (reviewed in Mercier and Hamel [19]). It would also seem to be maladaptive to use salinity as a cue for gamete release as low salinity has been shown to have detrimental effects on osmotic balance in the eggs of crown-of-thorns starfish, resulting in reduced cleavage and gastrulation rates [45]. Consistent with our results, less than 5% of green sea urchins (*Strongylocentrotus droebachiensis*) and blue mussels (*Mytilus edulis*) responded to addition of f/2 culture medium in the absence of phytoplankton [42]. These results suggest that water quality parameters (low salinity, high nutrients) typically associated with high phytoplankton abundance [38] do not directly induce spawning in crown-of-thorns starfish.

Frequency of spawning in male starfish in response to the three phytoplankton species tested was not significantly above control levels and none of the females spawned. This is consistent with results of work on the sea urchin, *Lytechinus variegatus*, where only a very small proportion of males and none of the females spawned in response to phytoplankton [46]. The duration of our experiment (720 min) may not have been enough to stimulate a significant spawning response, although phytoplankton cues must be detected on the onset of blooms for it to be advantageous to planktotrophic larvae since these events are often short-lived. We also cannot rule out that spawning response of crown-of-thorns starfish may be dependent on the concentration of phytoplankton, as previously shown for *S*. *droebachiensis* and *M*. *edulis* [42]. Nevertheless, phytoplankton concentrations used in this study were higher than concentrations that induced maximum spawning in experiments by Starr et al. [42] and maximum phytoplankton abundances from flood plume samples in the GBR [38]. In addition, mass spawning by crown-of-thorns starfish has also been observed in the absence of peaks in phytoplankton abundance in the GBR [12]. Although putative cues isolated from phytoplankton were found to be present in a variety of algal species, it is worth noting that *Skeletonema* induced 62.5% of males to spawn, compared to only 25% and 12.5% when exposed to *Chaetoceros* and *Dunaliella*, respectively. This variation may indicate a qualitative difference in the exudates of this microalgae species. Monitoring of flood plumes in the GBR has shown that elevated chlorophyll-*a* concentrations during high flow events are associated with the highest phytoplankton abundances, driven predominantly by high counts of nanoplankton species, particularly the diatoms *Skeletonema*, and *Chaetoceros* [38]. Further studies are warranted on the possible role on synchronous spawning of these abundant diatoms associated with flood plumes in the GBR. For echinoderms with planktotrophic larvae, such as crown-of-thorns starfish, it would be advantageous to time gamete release when environmental conditions are favorable for larvae [42,44,63]. However, apart from phytoplankton blooms induced by nutrient enrichment, flood events are also associated with environmental stressors, such as reduced salinity, which may have maladaptive consequences for gametes, fertilization, and embryonic development in crown-of-thorns starfish [45].

The presence of sperm or chemical cues associated with sperm and/or spawning induced gamete release in a large proportion of male and female starfish. In an aggregation of the sea urchin, *Sphaerechinus granularis*, one-third of the group immediately spawned after gamete release was induced in an individual and sea urchins downstream also started shedding gametes within 20 minutes [47]. Our results are also consistent with spawning induction assays where conspecific sperm triggered gamete release in *L*. *variegatus* [46]. Previous laboratory experiments have shown that pheromones extracted from ovaries and testes of crown-of-thorns starfish attract movement towards the spawning individual and triggers synchronous spawning among neighboring starfish [48]. Miller [49] also demonstrated that female starfish (*Asterias forbesi* and *Orthasterias koehleri*) produced long-lived sperm chemoattractants and proposed a model where males respond by migrating towards females and as the concentration of attractants increases (through aggregation and increased production by females with ripening ovaries), males are induced to spawn, releasing sperm that stimulates spawning in females. This is further supported by the finding that homogenates of ovaries from the brittle stars, *Ophiocoma dentata* and *Ophiocoma scolopendrina*, induced spawning in conspecific males, while sperm did not elicit any response [64]. Our results, however, show that eggs in the water column did not induce a significant proportion of starfish to release gametes. Combining sperm and phytoplankton did not increase the likelihood of spawning in both males and females, but it did slightly reduce the spawning response time in male starfish when compared to sperm treatments. This warrants further studies as sperm and phytoplankton have been shown to have synergistic effects in sea urchin spawning assays [42,46].

**Fig 3:**
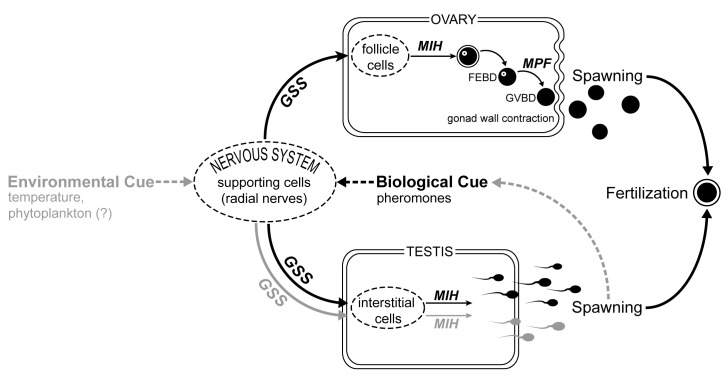
Schematic diagram of proposed cascade model for spawning induction and synchrony in response to environmental and biological cues. Grey arrows are responses to environmental cues and black arrows refer to biological cues. Neurohormonal mechanisms are based on Giese and Kanatani [14] and Mita et al. [15]. GSS = gonad-stimulating substance (relaxin-like gonad stimulating peptide); MIH = maturation-inducing hormone; MPF = maturation-promoting factor; FEBD = follicular envelop breakdown; GVBD = germinal vesicle breakdown.

Across all experiments, males were more likely to spawn in response to potential cues tested compared to females; and even if the females did spawn, males responded much faster. Sexual dimorphism in spawning has been reported in numerous broadcast spawning marine invertebrates, and in most cases, males initiate spawning before females [51]. This pattern is consistent with observations of *in situ* spawning by crown-of-thorns starfish, where some males initiate spawning followed by gamete shedding by females and other males (reviewed in Pratchett et al. [1]), albeit with some exceptions (see Babcock and Mundy [22]). If sperm is limited, females will most likely spawn first and induce males to spawn so that sperm dilution is minimized [64]. Alternatively, when sperm competition exerts a strong selective pressure, males typically spawn earlier to reach unfertilized eggs first [52]. Some males in a given population may be more sensitive to exogenous cues and gamete shedding by these males subsequently causes the release of pheromones that induce spawning in conspecifics [13,47]. Delay in female spawning may reflect constraints on the mechanism of egg release compared to sperm release in males, as it has to go through maturation ([14]; **Fig 3**). When placed in seawater with 1-methyladenine, testes tend to shed sperm immediately, while ovaries take 30–60 min [3]. *In situ* spawning experiments using the red sea urchin, *Strongylocentrotus franciscanus*, show that early-spawning males gained higher average fertilization, more extensive spatial cover of fertilization, and far fewer cases of reproductive failure compared to males that spawned later [52]. The delay in spawning by females may allow males to accumulate sperm to a critical concentration and eggs are not shed until this threshold sperm concentration in the water column is reached [51]. The optimal interval between the initiations of male and female spawning is influenced by flow conditions and the degree of sperm competition and aggregation [52]. Sperm of crown-of-thorns starfish has been shown to age more rapidly than eggs and must come in contact with eggs within 2 h from release to avoid wastage and fertilization failure [54].

The proportion of starfish spawning in response to the cues tested in this study may be comparable to the proportion of spawning observed in the field. For example, the most substantial natural spawning observed at Davies Reef involved only 60% of all individuals, but gamete density was enough to significantly reduce water visibility (R. Babcock, pers. comm.; [13]). Taken together, our experiments suggest that male crown-of-thorns starfish initiate spawning in response environmental cues (e.g. temperature change), which subsequently synchronizes spawning by inducing females and other males to spawn via biological cues (pheromones) from sperm in the water column (**Fig 3**). We propose that environmental cues act as spawning ‘inducers’ by causing the release of hormones (gonad stimulating substance) in sensitive males. Biological cues (pheromones: [48,49]) from released sperm, in turn, act as spawning ‘synchronizers’ by triggering a hormonal cascade resulting in gamete shedding by conspecifics. The ultimate environmental cue that induces gamete release remains unclear. Other environmental cues that were not tested here, such as length of photoperiod, light intensity, tides, and currents could also play a role in spawning induction [19]. Here we showed that an abrupt rise in temperature, rather than a defined threshold temperature, triggered spawning in male starfish. Majority of males also spawned in response to the presence of the diatom, *Skeletonema*, which is known to be abundant during high flow events in the GBR [38]. Marine invertebrates may use a hierarchy or combination of environmental cues to trigger synchronous spawning in a population (e.g. [65,66]). Crown-of-thorns starfish have been observed to participate in synchronous multi-specific spawning events in the GBR [12] and may respond to a common spawning signal released by other species that are shedding gametes. It is difficult to separate the stimuli for gametogenesis from the actual spawning cue, since the culmination of gamete production may itself stimulate spawning, as the pressure of gravid gonads may stimulate the gonadal musculature, thereby exciting the hormonal mechanisms [14]. Our experiments were conducted with isolated individuals, and the degree of synchrony might increase further if starfish were in close contact, so that cues could accumulate and be magnified among individuals. In comparing spawning between dispersed and aggregated populations, Okaji [67] suggested that aggregated individuals receive spawning stimuli at a higher frequency and magnitude compared to dispersed individuals, thereby accounting for better synchronization and higher reproductive output. Spawning was also minimal in small populations of *S*. *droebachiensis* compared to a large and dense population, implying that sperm concentration may not have been high enough to trigger pheromone-mediated spawning in less responsive urchins [66]. Differences in the physiological condition of individuals and temporal or spatial variation in the concentration or magnitude of environmental cues may also explain the unpredictability of crown-of-thorns starfish spawning events. Given the immediate temporal linkage between the timing of spawning and fertilization events, variability in the extent and synchronicity of gamete release may significantly influence reproductive success and explain marked fluctuations in the abundance and distribution of crown-of-thorns starfish populations.

## Supporting information

S1 Table Odds ratios and confidence intervals of pairwise comparisons between treatments for each spawning experiment. (DOCX)
