# Supplementary material for: Environmental and biological cues for spawning in the crown-of-thorns starfish
Source: PLoS One. 2017 Mar 29;12(3):e0173964. doi: 10.1371/journal.pone.0173964 (PMC5371309; doi:10.1371/journal.pone.0173964)
Supplement: S1 Table — (DOCX) [file pone.0173964.s001.docx]

**S1 Table.** Odds ratios and confidence intervals of pairwise comparisons between treatments for each spawning experiment. FSW = 0.2-μm filtered seawater; LS-FSW = low salinity filtered seawater; NE-FSW = nutrient-enriched filtered seawater; PP = combination of three phytoplankton species.

| **SOURCE** | **ODDS RATIO** | **95% CI** | **p-value** |
| --- | --- | --- | --- |
| **(a)** TEMPERATURE |  |  |  |
| *Male* |  |  |  |
| 28°C vs 28°C🡪30°C | 3.667 | (0.118 – 113.736) | 1.000 |
| 28°C vs 26°C🡪30°C | 121.000 | (2.017 – 7259.723) | **0.008** |
| 28°C🡪30°C vs 26°C🡪30°C | 33.000 | (1.064 – 1023.620) | **0.048** |
| *Female* |  |  |  |
| 28°C vs 28°C🡪30°C | 1.000 | (0.017 – 59.998) | 1.000 |
| 28°C vs 26°C🡪30°C | 3.667 | (0.118 – 113.736) | 1.000 |
| 28°C🡪30°C vs 26°C🡪30°C | 3.667 | (0.118 – 113.736) | 1.000 |
| **(b)** WATER QUALITY |  |  |  |
| *Male* |  |  |  |
| FSW vs LS-FSW | 1.923 | (0.197 – 18.812) | 1.000 |
| FSW vs NE-FSW | 1.000 | (0.084 – 11.932) | 1.000 |
| LS-FSW vs NE-FSW | 1.923 | (0.197 – 18.812) | 1.000 |
| *Female* |  |  |  |
| FSW vs LS-FSW | 1.000 | (0.018 – 56.466) | 1.000 |
| FSW vs NE-FSW | 1.000 | (0.018 – 56.466) | 1.000 |
| LS-FSW vs NE-FSW | 1.000 | (0.018 – 56.466) | 1.000 |
| **(c)** PHYTOPLANKTON |  |  |  |
| *Male* |  |  |  |
| Control vs *Dunaliella* | 1.000 | (0.084 – 11.932) | 1.000 |
| Control vs *Skeletonema* | 7.857 | (0.865 – 71.385) | 0.119 |
| Control vs *Chaetoceros* | 1.923 | (0.197 – 18.812) | 1.000 |
| *Dunaliella* vs *Skeletonema* | 7.857 | (0.865 – 71.385) | 0.119 |
| *Dunaliella* vs *Chaetoceros* | 1.923 | (0.197 – 18.812) | 1.000 |
| *Skeletonema* vs *Chaetoceros* | 4.086 | (0.564 – 29.617) | 0.315 |
| *Female* |  |  |  |
| Control vs *Dunaliella* | 1.000 | (0.018 – 56.466) | 1.000 |
| Control vs *Skeletonema* | 1.000 | (0.018 – 56.466) | 1.000 |
| Control vs *Chaetoceros* | 1.000 | (0.018 – 56.466) | 1.000 |
| *Dunaliella*l vs *Skeletonema* | 1.000 | (0.018 – 56.466) | 1.000 |
| *Dunaliella* vs *Chaetoceros* | 1.000 | (0.018 – 56.466) | 1.000 |
| *Skeletonema* vs *Chaetoceros* | 1.000 | (0.018 – 56.466) | 1.000 |
| **(d)** GAMETE |  |  |  |
| *Male* |  |  |  |
| Control vs Sperm | 44.200 | (1.795 – 1088.207) | **0.007** |
| Control vs Egg | 3.400 | (0.120 – 96.706) | 1.000 |
| Sperm vs Egg | 13.000 | (1.329 – 127.168) | **0.041** |
| *Female* |  |  |  |
| Control vs Sperm | 10.818 | (0.463 – 252.804) | 0.200 |
| Control vs Egg | 3.400 | (0.120 – 96.706) | 1.000 |
| Sperm vs Egg | 3.182 | (0.350 – 28.908) | 0.569 |
| **(e)** SPERM and PP |  |  |  |
| *Male* |  |  |  |
| Control vs Sperm | 26.714 | (1.143 – 624.270) | **0.026** |
| Control vs Sperm + PP | 26.714 | (1.143 – 624.270) | **0.026** |
| Sperm vs Sperm + PP | 1.000 | (0.150 – 6.655) | 1.000 |
| *Female* |  |  |  |
| Control vs Sperm | 10.818 | (0.463 – 252.804) | 0.200 |
| Control vs Sperm + PP | 6.538 | (0.266 – 160.977) | 0.467 |
| Sperm vs Sperm + PP | 1.655 | (0.228 – 11.994) | 1.000 |

* Fishers Exact Test p-value of pairwise comparisons
